# Acupoint catgut embedding for the treatment of peptic ulcers: A protocol for systematic review and meta analysis

**DOI:** 10.1097/MD.0000000000025562

**Published:** 2021-04-23

**Authors:** Xingqian Yi, Li Wang, Qingying He, Rigun A, Yimin Le

**Affiliations:** a Jiangxi University of Traditional Chinese Medicine; b Science and Technology College of Jiangxi University of Traditional Chinese Medicine, Nanchang, China.

**Keywords:** Acupoint catgut embedding, peptic ulcers, protocol, systematic review

## Abstract

**Background:**

Peptic ulcer (PU) is a common clinical disease of the digestive system, which can occur in all ages, gastric and duodenal ulcers are the most commonly seen PUs in clinical practice. The main manifestations are chronic and periodic rhythmic upper abdominal pain, accompanied by indigestion symptoms such as pantothenic acid, belching, and nausea. Serious complications such as bleeding, perforation, obstruction and canceration are easy to occur, endangering the life safety of patients. There are many ways to treat PU in clinic, and acupoint catgut embedding therapy has its unique advantages. Hence, our systematic review aims to evaluate the efficacy and safety of acupoint embedding therapy in the treatment of PU and to provide a reliable basis for physician.

**Methods:**

We will search electronic databases including PubMed, Embase, Cochrane Library, China National Knowledge Infrastructure (CNKI), Wanfang Database (WF), China Biomedical Literature Database (CBM), and China Scientific Journals Database (VIP) from establishment to April 2021, and will manually searched the list of medical journals as a supplement. Two authors will screen the studies independently, as well as extract data information, and assess methodological quality through the Cochrane risk of bias (ROB) tool. The Stata software (Version 16.0) software will be used for statistical analysis.

**Results:**

By evaluating the current status of acupoint catgut embedding for Peptic ulcer disease, this study would prove the effectiveness and safety of acupoint embedding therapy, and will be published in a peer-reviewed journal.

**Conclusion:**

This systematic review will provide a credible evidence-based for acupoint catgut embedding in the treatment of peptic ulcer.

**INPLASY Registration number:**

INPLASY202130097

## 1 Introduction

Peptic ulcer is mainly refers to the gastric ulcer and duodenal ulcer,^[1]^ which is a common and frequently-occurring disease of the digestive system disease, occurs in all age groups.^[2]^ Modern medicine believes that the occurrence of the disease is related to many factors, such as *Helicobacter pylori* infection, Non-steroidal anti-inflammatory, abnormal gastric acid secretion stress and weakening of gastric mucosa protective barrier.^[3–6]^ Among them, the *H. pylori* infection is considered to be the main cause of ulcer disease, which is often induced by fatigue, poor mood, improper diet and drug stimulation. The main clinical manifestations are epigastric pain, dysphoria and other symptoms, which have the characteristics of chronic, periodic and rhythmic attacks, which seriously affect the quality of life of patients.^[7,8]^ At present, acid suppression, eradication of *H. pylori* (eg, triple therapy, quadruple therapy, sequential therapy, etc.) and gastric mucosal protection are often used in treatment.^[9,10]^ The short-term effect is remarkable, but the recurrence rate is high,^[11]^ and elderly patients are prone to malignant change after recurrence. Along with the development of society, the incidence of peptic ulcer is a growing trend and patients increased gradually in young adults.^[12]^ Effective prevention and treatment of digestive system diseases has become a practical problem to be solved urgently.

Traditional Chinese medicine (TCM), especially acupuncture, and integrated medicine are the current research focus on the treatment of PU.^[13]^ Studies have shown that acupuncture can regulate viscera, reconcile qi and blood, dredge channels and collaterals, activate blood circulation and remove blood stasis, promote ulcer healing and protect gastric mucosa.^[14]^ However, the effect of acupuncture alone in the treatment of chronic intractable diseases is often not satisfactory, or although it is effective, it can not be consolidated and lasting.^[2]^ Acupoint catgut embedding therapy, as the extension and development of acupuncture therapy, is a compound treatment method which integrates many kinds of effects, such as acupoint stimulation effect, pricking blood effect, biological reaction and so on.^[15]^ Through the biophysical function and biochemical changes of catgut in acupoint, its stimulating information and energy are transferred into the body from meridians and collaterals, so as to achieve the purpose of “soothing its blood gas and making it reach”. Catgut embedding therapy has a wide range of indications,^[16]^ especially for painful diseases, functional diseases and chronic diseases.^[17]^ it not only has the characteristics of replacing acupuncture with thread, double effect of acupuncture and medicine, lasting stimulation and stable curative effect,^[18]^ but also can reduce the frequency and medical expenses of patients. it has a unique advantage in improving the dependence of patients.^[19–21]^ Digestive ulcer is a chronic and recurrent disease. Through its long-lasting and mild acupuncture-like stimulation, catgut embedding at acupoint can dredge meridians, smooth qi and blood, coordinate viscera and restore long-term benign stimulation of qi rise and fall, which is conducive to the healing of ulcers.^[22]^ Catgut embedding can also adjust or enhance gastric secretion to control the amount of gastric acid secretion,^[23]^ adjust the imbalance between yin and yang and viscera function, and promote mucosal healing.^[24]^ It is widely used in clinic and has achieved good curative effect.^[25,26]^ However, there is still a lack of systematic review and meta-analysis on the efficacy and safety of acupoint catgut embedding therapy in the treatment of peptic ulcer. Hence, we will systematically review and meta-analyze the efficacy and safety of clinical randomized controlled trials of acupoint catgut embedding in the treatment of PU. Where possible, short-term and long-term results of single use, combination of western medicine or traditional Chinese medicine will be evaluated, in order to provide a reliable basis for clinical decision-makers.

## 2 Methods

### 2.1 Inclusion criteria

#### 2.1.1 Types of studies

RCTs assessing acupoint catgut embedding treatment for PU will be eligible for inclusion and were published in English or Chinese, with the full-text available.

#### 2.1.2 Types of participants

Patients meet the diagnostic criteria of PU will be included. No race, age, gender, nationality and source of cases limited. Participants who with complications of peptic ulcer, digestive tract tumor, reflux esophagus, mental illness, cardio-cerebrovascular, liver, kidney and hematopoietic system and other serious primary diseases will be excluded.

#### 2.1.3 Types of interventions

Acupoint catgut embedding therapy as a single intervention for PU in clinical will be include, or combined with other intervention (eg, conventional drugs, Chinese herbs, acupuncture, etc.).

#### 2.1.4 Types of control group

The treatment of the control group has no restrictions, including conventional drugs, no treatment, or placebo.

#### 2.1.5 Types of outcome measures

##### 2.1.5.1 Primary outcome

Primary outcomes mainly include the total effective rate, Hp eradication rate, recurrence rate of ulcers.

##### 2.1.5.2 Additional outcomes

The adverse reactions, electronic gastroscopy ulcer healing rate and clinical symptom score will be regarded as an additional result.

### 2.2 Search methods for the identification of studies

We will search electronic databases including PubMed, Embase, Cochrane Library, China Biomedical Literature Database (CBM), China National Knowledge Infrastructure (CNKI), Wanfang Database (WF), and China Scientific Journals Database (VIP) to collect potential RCTs from establishment to April 2021. Search terms of disease: peptic ulcer, digestive ulcer, digestion ulcer, Peptic ulcer disease, gastric ulcer, stomach ulcer, duodenal ulcer; and intervention, acupoint catgut embedding, catgut embedding, catgut embedding therapy, catgut implantation, point embedding therapy; and research type: randomized controlled trial, controlled clinical trial, randomized. The PubMed search strategy is shown in Table 1

**Table 1:** Search strategy (PubMed database).

Number	Search items
#1	Mesh: “Peptic ulcer”
#2	Ti/Ab: “Ulcer” OR “peptic ulcer” OR “gastric ulcer” OR “duodenal ulcers” OR “digestibility ulcer” OR “peptic colitis” OR “Crohn's disease”
#3	#1 OR #2
#4	Mesh: “Catgut” OR “embryo implantation”
#5	Ti/Ab: “Catgut” OR “embryo” OR “implantation” OR “embryo implantation” OR “acupuncture” OR “points” OR “acu-points” OR “thread” OR “thread embedding” OR “acupuncture therapy”
#6	#4 OR #5
#7	Mesh: “randomized controlled trial [Publication Type]” OR “controlled clinical trial [Publication Type]”
#8	Ti/Ab: Randomized OR Trial OR Contrast OR Groups
#9	#7 OR #8
#10	#3 AND #6 AND #9

### 2.3 Data collection and analysis

#### 2.3.1 Selection of studies

**Figure 1:**
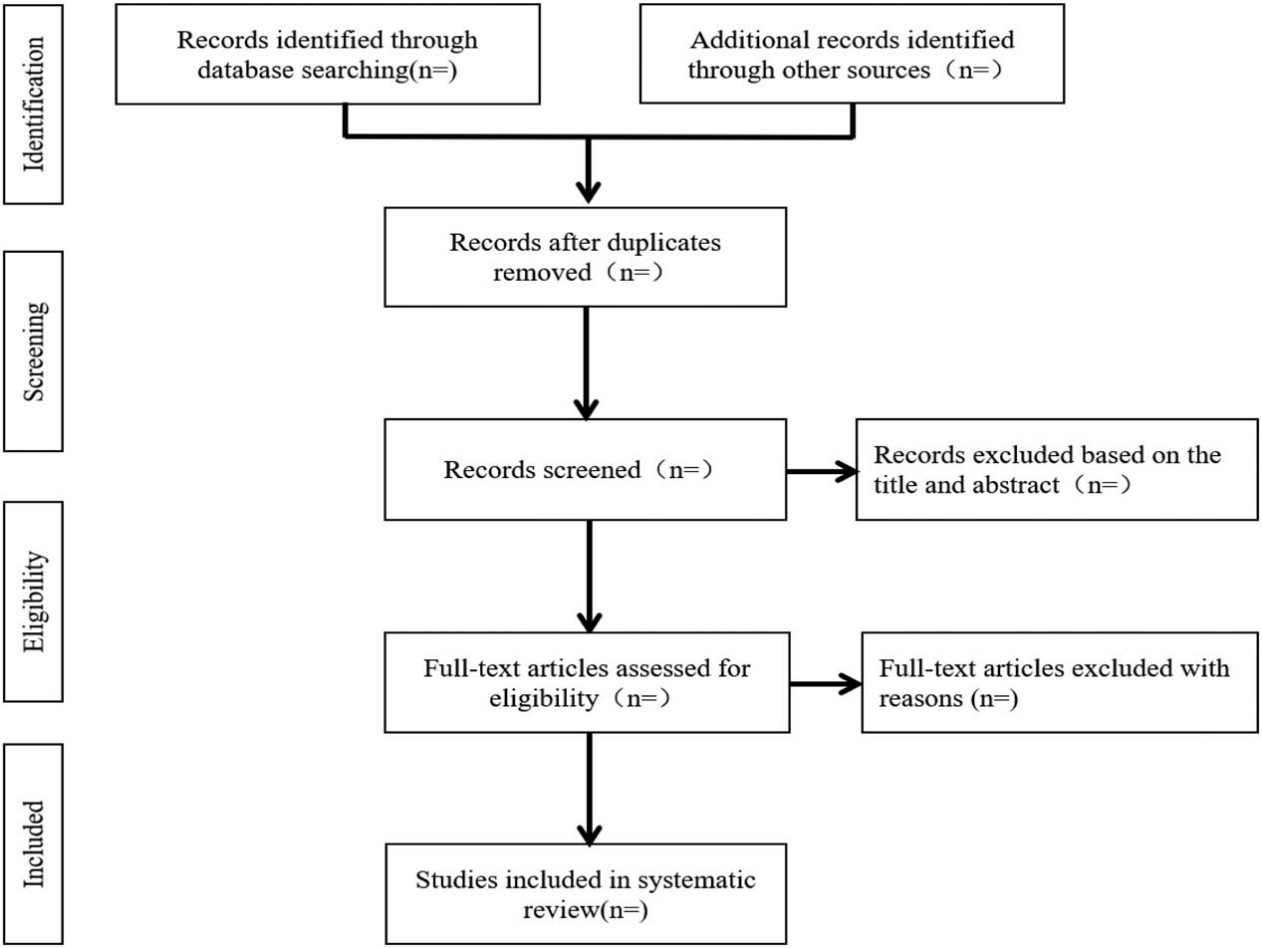
Flow diagram of study selection process.

We will import the retrieved results into EndNote X7 software and delete the duplicate data. After that, two authors will independently scan the titles, abstracts and full texts of the literature according to the inclusion and exclusion criteria to evaluate the eligibility of these articles. Any different opinions will be resolved by the third author. The study selection procedure is summarized in Figure 1.

#### 2.3.2 Data extraction and management

Two reviewers will independently extract relevant data from the eligible RCTs, including the first author, participants’ baseline characteristics, sample size, intervention, intervention time, follow-up, results, and adverse events. Any discrepancies will be resolved through consultation with a third reviewer. If necessary, we will also contact the original author for more information.

### 2.4 Risk of bias assessment

The risk of bias (ROB) assessment tool recommended by Cochrane Collaboration Network was used to evaluate the quality of the included studies. Including the following 7 evaluation items:

1. random sequence generation;
2. allocation concealment;
3. blinding of participants and personnel;
4. blinding of outcome assessment;
5. incomplete outcome data;
6. selective reporting;
7. other sources of bias. For each study, the results were rated as “Yes” (low risk), “No” (high risk) and “unclear” (lack of relevant information or uncertainty about bias) for above seven items.

Two reviewers independently performed quality assessment and all disagreements will be resolved by discussion.

### 2.5 Quantitative data synthesis and statistical methods

#### 2.5.1 Quantitative data synthesis

We will use the Stata software (Version 16.0) software for statistical analysis. For continuous variables, when outcomes were measured by the same scale, the results were reported as standardized mean difference (MD) and 95% confidence interval (CI); when different scales were used, the results were reported as standardized mean difference (SMD) and 95% CI. Categorical data will be calculated with the risk ratio (RR) and 95% CI.

#### 2.5.2 Assessment of heterogeneity

We will use I^2^ test and Chi-square test to evaluate the heterogeneity of the results. When I^2^ ≤ 50% and *P* > .10, the results of the study will be considered as homogeneous, and fixed effect model will be used; otherwise, random effect model will be used.

#### 2.5.3 Subgroup analysis

If significant heterogeneity is detected in our meta-analysis, we will perform subgroup analysis based on different control groups.

#### 2.5.4 Sensitivity analysis

When there are sufficient RCTs, we will conduct sensitivity analysis based on methodological quality, sample size and missing data to evaluate the robustness of the research results.

#### 2.5.5 Assessment of reporting biases

Publication bias will be analyzed through the funnel plot. If the funnel plot is asymmetric, there may be a publication bias in the research results.

## 3 Discussion

At present, the efficacy and safety of acupoint catgut embedding in the treatment of obesity, diarrhea, diabetes and other diseases have been reported, but as far as we know, this is the first time for peptic ulcer. The conclusion of this study will expand the treatment scope of acupoint catgut embedding therapy. Provide evidence-based medicine advice for the treatment of peptic ulcer, and more and better treatment options for ulcer patients. However, our conclusions may have potential limitations. First, acupoint selection, different materials of catgut, severity of illness, treatment frequency and different control groups may lead to potential heterogeneity. Heterogeneity must be explained by subgroup analysis or sensitivity analysis. Secondly, the measurement and tools of included randomized controlled trials may be different, which increases the difficulty and complexity of subgroup analysis and may affect the evaluation results. Finally, we only included the Chinese and English articles, the other language articles are overlooked.

## Author contributions

**Data curation:** Xingqian Yi, Li Wang.

**Formal analysis:** Xingqian Yi, Li Wang.

**Investigation:** Xingqian Yi, Li Wang.

**Methodology:** Qingying He, Rigun A.

**Project administration:** Xingqian Yi, Yimin Le.

**Software:** Qingying He, Li Wang.

**Supervision:** Yimin Le.

**Validation:** Yimin Le.

**Writing – original draft:** Xingqian Yi, Yimin Le.

**Writing – review & editing:** Xingqian Yi, Yimin Le.
